# Transversus abdominis plane block with different bupivacaine concentrations in children undergoing unilateral inguinal hernia repair: a single-blind randomized clinical trial

**DOI:** 10.1186/s12871-022-01907-y

**Published:** 2022-11-21

**Authors:** Meltem Savran Karadeniz, Ayşe Gülşah Atasever, Emine Aysu Salviz, Emre Sertaç Bingül, Hayriye Şentürk Çiftçi, Müşerref Beril Dinçer, Mukadder Orhan Sungur

**Affiliations:** 1 Department of Anesthesiology and Intensive Care, Istanbul University Istanbul Faculty of Medicine, Istanbul, Turkey; 2 Department of Anesthesiology, University Hospitals of the KU Leuven, Herestraat 49, 3000 Leuven, Belgium; 3 Department of Anesthesiology, Washington University School of Medicine, St. Louis, MO USA; 4 Department of Medical Biology, Istanbul University Istanbul Faculty of Medicine, Istanbul, Turkey

**Keywords:** Transversus abdominis plane block, Regional anesthesia, Pediatrics, Inguinal hernia repair

## Abstract

**Background:**

Current knowledge on the ideal local anesthetic concentration for the ultrasound-guided transversus abdominis plane block (TAPB) in pediatrics is scarce. The purpose of this study is to compare the efficacy of US-guided TAPB at two different concentrations of bupivacaine in pediatrics undergoing unilateral inguinal hernia repair.

**Methods:**

After random allocation, 74 children aged 1–8 were randomized to receive US-guided TAPB by using 1 mg.kg^− 1^ bupivacaine as either 0,25% (0,4 ml.kg^− 1^) (Group 1) or 0,125% (0,8 ml.kg^− 1^) (Group 2) concentration. All blocks were performed under general anesthesia, immediately after the induction, unilaterally with a lateral approach. All subjects received intravenous 15 mg/kg paracetamol 0.15 mg/kg dexamethasone and 0.1 mg/kg ondansetron intraoperatively. The primary outcome was the efficacy which is assessed by postoperative FLACC behavioral pain assessment score at 15′, 30′, 45′, 1 h, 2 h, 6 h, and 24 h. The secondary outcomes were to assess the total dose of rescue analgesic consumption, length of hospital stay, the incidence of side effects, complications and satisfaction levels of the patients’ parents and the surgeons.

**Results:**

Sixty-four children were recruited for the study. Postoperative pain scores were equal between the two groups. There was no need for a rescue analgesic in any group after the postoperative 6^th^hour. No local or systemic complication or side effect related to anesthesia or surgery was reported.

**Conclusion:**

TAPB using 1 mg.kg^− 1^ bupivacaine administered as either high volume/low concentration or low volume/high concentration was providing both adequate analgesia and no side effects.

**Trial registration:**

This trial was retrospectively registered at Clinicaltrals.gov, NCT04202367.

**Supplementary Information:**

The online version contains supplementary material available at 10.1186/s12871-022-01907-y.

## Introduction

Optimal postoperative analgesia in children is of utmost importance as they are more susceptible to narcotics. Abdominal surgery is associated with varying degrees of incisional and visceral pain that benefits from optimal analgesia in the perioperative period [1]. Therefore, transversus abdominis plane block (TAPB) has gained popularity to provide opioid-sparing analgesia as a part of a multimodal approach during the last decade. This block provides analgesia by blocking the 10th and 11th intercostal nerves (T10–T11), the subcostal nerve (T12), and the ilioinguinal nerve and iliohypogastric nerve (L1) depending on the injection site [2–4]. It implies a single-shot injection of a local anesthetic solution in the fascia layer between the internal oblique and the transversus abdominis muscle. Typically, a distinct neural structure is not seen in this fascial plane. Instead, there is an extension of small nociceptive fibers within the intended target to anesthetize [5].

The procedure promotes excellent analgesia to the parietal peritoneum as well as the skin and muscles of the anterior abdominal wall. The concentration and the dose of local anesthetics (LA) have particular importance for the success of pain control. Recent data from randomized controlled trials (RCTs) showed improved clinical efficacy of TAPB when using ultrasound (US) compared with other common pain control techniques [2, 6–9]. The TAPB is an interfascial plane block; therefore, the success of the block depends on the volume of local anesthetic injected. However, minimal effective volume for TAPB has not been defined yet in children [10, 11]. The use of a large amount of local anesthetic may make the analgesic effect more reliable. On the other hand, children require more caution towards increased LA doses. Therefore, it is still needed to investigate further to describe effective amounts of LA doses for TAPB in children [12].

Theoretically, low-concentrated LA mixtures prevent neurotoxicity with suspicion of less analgesic efficacy. Therefore, in order to exhibit clinical implications, we hypothesized that an ipsilateral US-guided TAPB with a high-volume low concentration (HVLC) bupivacaine solution would promote better pain control than a low-volume high concentration (LVHC) solution. The primary outcome of this study was to compare the postoperative pain scores up to the first 24 hours. The secondary outcomes were to assess the percentage of patients receiving rescue analgesics on the first postoperative day, the total dose of rescue analgesic consumption, length of hospital stay, the incidence of side effects and complications, and satisfaction levels of the patients’ parents and the surgeons.

## Materials and methods

### Study design and study subjects

The present study is a prospective, single-blind, RCT, performed according to the principles of good clinical practice and the International Declaration of Helsinki. The study was approved by the Ethics Committee of Istanbul University, Istanbul Faculty of Medicine, Istanbul (dossier no: 1281/2016). The study is retrospectively registered on Clinicaltrials.gov (Ref. NCT04202367, first registration date was 17/12/2019.) and reported according to the CONSORT statement.

After obtaining written informed parental consent, 74 (37 in group 1 and 37 in group 2) children were allocated to this trial. Children aged between 1 and 8 years in the ASA I and II, undergoing unilateral open inguinal hernia repair were eligible for the study. Children aged less than 1 or more than 8 years; those with a neurological deficit, bleeding diathesis, or a history of allergy to local anesthetics; children whose physical examination revealed an infection in the region to be injected; those who have mental retardation or communication problems; and subjects who did not accept to participate in the study were excluded.

### Patient randomization

After inclusion/exclusion criteria were met, randomization was performed using a web-based system that generates numbers for the participants (www.graphpad.com/quickcalcs/randMenu/). Allocation numbers were presented in sealed opaque envelopes, which were prepared by an independent researcher before the start of the study, and these envelopes were opened in the operating room by the anesthetist on the day of surgery. This investigation was planned as single-blinded. Since the LA doses were visibly distinguishable, blindness could not be provided by the operating anesthetist. However, clinical follow-up data were collected by researchers who were blinded to the dosage and volume of the LA.

### Anesthesia, intervention, and post-interventional follow-up

Patients were monitored with standard ASA recommendations including pulse oximetry, non-invasive blood pressure, end-tidal CO_2_, temperature, and electrocardiogram. Patients were premedicated, 30 minutes prior to the surgery, with oral midazolam 0,5 mg.kg^− 1^ once diluted in apple juice. For the induction of anesthesia, sevoflurane 4–6% was administered via a face mask. Subsequently, intravenous rocuronium (0,3–0,6 mg.kg^− 1^) was administered and a laryngeal mask was installed, and TAPB was performed prior to incision.

To perform the lateral approach; the iliac crest, 12th rib and midaxillary line were identified as the landmarks, and the in-between area was disinfected using 2% chlorhexidine in 70% alcohol [10]. A high frequency (7–15 MHz) linear ultrasound probe (GE Logiq-e Nextgen model, General Electric medical systems, Phoenix, AZ, USA) was covered with a sterile sheath, and placed on the midaxillary line transversally between arcus costarum and iliac crest. Once the muscular fascia between the internal oblique and the transversus abdominis muscle was identified, a peripheric block needle (22G, 50 mm, Stimuplex®; B. Braun, Melsungen, Germany) was advanced in-plane from anterior to posterior aiming for the interfascial space. Needle tip placement was checked via 0.5 ml saline injection, and if the desired fascial expansion was observed, the prepared LA solution was injected [4, 13].

A US-guided TAPB was provided in equal doses (1 mg.kg^− 1^) but with different concentrations of bupivacaine on the ipsilateral side of the incision.

**Group 1:** Patients received 1 mg.kg^− 1^bupivacaine 0.25%, 0.4 ml.kg^− 1^.

**Group 2:** Patients received 1 mg.kg^− 1^bupivacaine 0.125%, 0.8 ml.kg^− 1^.

Anesthesia was maintained with sevoflurane 2% in oxygen (FiO_2_ = 0.35) using a closed-circuit respirator and intravenous 0.1 μg.kg^− 1^.min^− 1^ remifentanil infusion. All subjects received paracetamol 15 mg.kg^− 1^ intraoperatively. Dexamethasone 0,15 mg/kg and ondansetron 0,1 mg/kg were given as a bolus for the prophylaxis of postoperative nausea and vomiting (PONV).

Postoperative pain was assessed by FLACC (Face, Legs, Activity, Cry, Consolability) behavioral pain assessment score at 15-, 30-, 45- minutes, and 1-, 2-, 6-, and 24-hour. Tramadol 1 mg.kg^− 1^ was administered intravenously as a rescue analgesic if the pain score was equal to or more than 4 at the post-operative care unit. The total analgesic requirement was recorded. All children were prescribed oral paracetamol 15 mg.kg^− 1^ four times per day for postoperative pain. All children were discharged on the same day after being comfortable, mobile, and tolerating oral fluids.

### Primary outcome

The primary outcome was the efficacy which is assessed by postoperative FLACC behavioral pain assessment score up to the first 24 hours. FLACC evaluation was taught to the caring legal guardians by the ward nurse in the preoperative period when the children were admitted to the ward. Acute postoperative period FLACC scores were recorded by the anesthesiology research team. Detailed information was obtained by calling the parents of the children who were discharged earlier than 24 hours.

### Secondary outcomes

The secondary outcomes included the percentage of patients receiving rescue analgesics on the first postoperative day, the cumulative dose of rescue analgesic consumption, length of hospital stay, the incidence of side effects including nausea, vomiting, hypotension, motor weakness, and urinary retention, complications (e.g., visceral puncture, vascular injury) and satisfaction of the patients’ parents and the surgeons. The satisfaction was questioned as “pleased” or “not pleased”.

### Statistical analysis

Sample size calculation was performed using G* Power version 3.1.9.2 (Kiel University, Kiel, Germany) software. According to our 10-patient pilot study, FLACC scores data at postoperative 6th hour (0.7 ± 0.2 for Group 1 and 0.5 ± 0.3 for Group 2), 27 patients per group were needed with a power of 0.8 while alpha was 0.05. Considering a 20% drop-out, a total number of 66 patients were planned to enroll in the study. The Shapiro-Wilk test method was used to test the normality of the continuous data. Baseline characteristics are presented as mean ± standard deviation or median (minimum-maximum) for quantitative variables, and as number (percentage) for qualitative variables. Categorical data were compared using a χ2 test with Fisher’s exact test, depending on sample sizes. For continuous outcomes, we compared groups using the Student’s t-test or Mann–Whitney U-test, depending on the data distribution. Data analysis was performed using SPSS 22 software (IBM Corp., Armonk, NY, USA). A *p*-value ≤0.05 was considered significant in outcome analysis.

## Results

### Study flow, baseline characteristics, procedural characteristics, and risk category

**Fig. 1:**
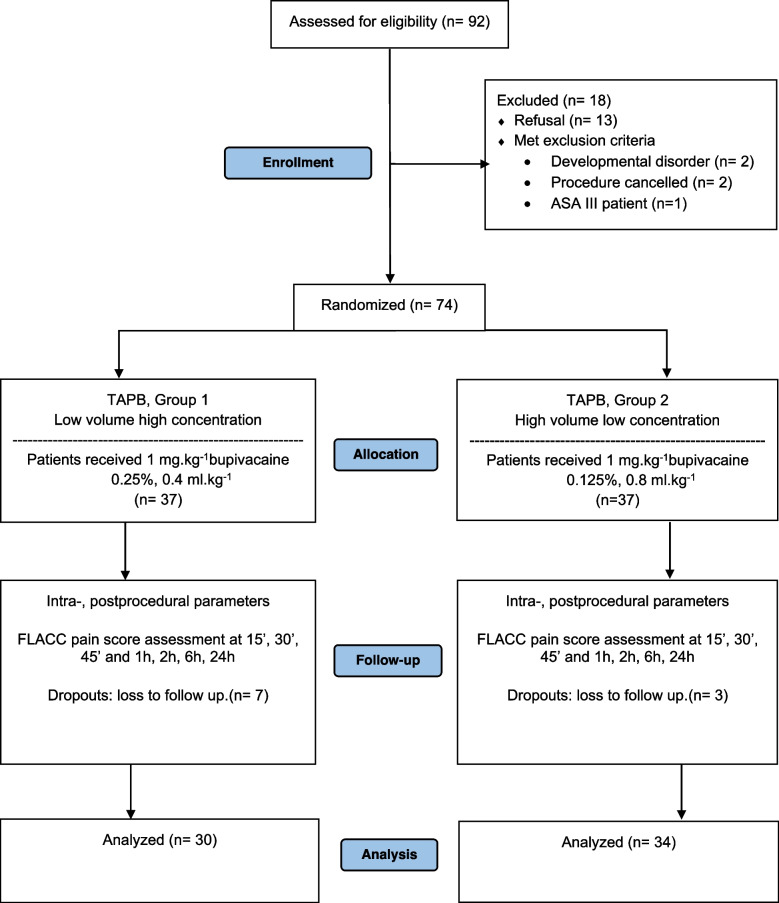
Flow diagram according to the CONSORT statement

From 01 November 2016 to 01 June 2017, 74 children aged between 1 and 8 years old were included in this trial and randomized to receive a TAPB with either 0,25% or 0,125% concentration of bupivacaine. Ten children (seven in group 1 and three in group 2) were excluded due to loss of follow-up. Therefore, 64 children were recruited for the study. The flowchart of the study cohort is summarized in Fig. 1. There were no differences in demographic or operative clinical parameters between the study groups (Table 1).

**Table 1:** Baseline demographic characteristics of subjects in both groups

	Group 1 (*n* = 30)	Group 2 (*n* = 34)	*p*-value
**Age, y**	**4,59 ± 2,48**	**4,17 ± 2,27**	**0.4**
**Sex, (m/f)**	**26(86,7%) /4(13,3%)**	**27(79,4%) /7(20,6%)**	**0.4**
**Height (cm)**	**113,42 ± 28,39**	**96,71 ± 29,70**	**0.1**
**Weight (kg)**	**21,13 ± 10,13**	**16,35 ± 6,41**	**0.05**
**Duration of anesthesia (min)**	**49,53 ± 11,89**	**43,97 ± 12,52**	**0.1**
**Duration of operation (min)**	**38,11 ± 10,69**	**34,88 ± 9,76**	**0.2**
**Rescue analgesia requirement n (%)**	**15 (50)**	**12 (35,3)**	**0.2**
**Total analgesic requirement (mg)**	**20 (9–55)**	**17 (9–30)**	**0.1**
**Length of hospital stay (h)**	**15,68 ± 8.58**	**11,72 ± 7,79**	**0.09**

Values are presented as mean ± standard deviation. Rescue analgesic requirement is presented as number of cases (%). Total analgesic requirement is presented as median (minimum -maximum). Categorical data were compared using a χ2 test with Fisher’s exact test, depending on sample sizes. For continuous outcomes, we compared groups using the Student’s t-test and Mann–Whitney U-test

### Primary outcome

Postoperative median pain scores were less than 4 at all-time points in all groups. Postoperative pain scores were equal between the groups at all time points (*p* > 0.05) (Table 2).

**Table 2:** The comparison of FLACC behavioral pain scores in two groups

FLACC score	Group 1 (***n*** = 30)	Group 2 (***n*** = 34)	*p*-value
15’	2 (0–7)	1 (0–6)	0.4
30’	1 (0–6)	2 (0–7)	0.9
45’	0 (0–5)	0 (0–5)	0.6
1 h	0 (0–6)	0 (0–4)	0.3
2 h	0 (0–1)	0 (0–3)	0.5
6 h	0 (0–4)	0 (0–3)	0.07
24 h	0 (0–2)	0 (0–1)	0.4

Values are presented as median (minimum-maximum). For continuous outcomes, we compared groups using the Mann–Whitney U-test

### Secondary outcomes

None of the patients need rescue analgesics after the postoperative 6th hour. The percentage of patients receiving rescue analgesics on the first postoperative day was similar between groups (*p* > 0.05). The cumulative rescue analgesic requirement was not significant between groups (*p* > 0.05). No local or systemic complication or side effect related to block or surgery was reported. The length of hospital stay was similar between the groups (*p* > 0.05). There was no difference among the groups in terms of parent and surgeon satisfaction levels.

## Discussion

The present study showed that ipsilateral TAPB with HVLC is equally effective as LVHC in controlling postoperative pain in inguinal hernia repair. In our study, the pain scores in recovery were similar between groups at all time points. Furthermore, the total number of patients requiring tramadol in recovery did not differ between the groups.

US-guided TAPB has gained popularity as part of multimodal analgesia in children in the last decade. Peripheral nerve blocks consist of 3 important elements: techniques, agents, and equipment. The ideal technique is embraced with the manner of US guidance and a predetermined endpoint for needle placement. Real-time two-dimensional US allows us to visualize the tip of the needle and local anesthetic spread towards the transversalis facial plane. The local anesthetic should be defined as quick onset, predictable spread, and high quality. The procedure should also be performed with the minimum risk of complications and side effects. In line with this purpose, we administered 1 mg.kg^− 1^ bupivacaine solution for all blocks with a single shot to capture all the nerves, extending down from the spinal cord. Similarly, a recent RCT by Sola et al. examined the optimal concentration of levobupivacaine for successful US-guided TAPB in children [14]. The authors showed that the quality of postoperative pain control provided by TAPB using levobupivacaine 0.4 mg.kg^− 1^ administered as either HVLC or LVHC did not differ and was associated with a very low risk of local anesthetic systemic toxicity.

Few publications have addressed the pharmacodynamics of local anesthetics in children [15, 16]. The latest European Society of Regional Anesthesia and Pain Therapy/American Society of Regional Anesthesia and Pain Medicine Recommendations confirms that the performance of US-guided fascial plane blocks (eg. rectus sheath, TAPB, fascia iliaca) can be performed successfully and safely using a recommended LA dose of bupivacaine or ropivacaine of 0.25 to 0.75 mg/kg (Evidence B1) [17]. Suresh et al. have evaluated two different bupivacaine doses in children and have shown a higher dose of local anesthetic is viable to improve analgesia in children after a US-guided TAPB [18]. Our results may be explained by the recommended doses of local anesthetic that we administered.

To date, a few RCTs have compared the efficacy of TAPB to other common pain control techniques in children, such as field blocks, and caudal epidural blocks. Sethi et al. showed that caudal epidural block provides a significantly prolonged duration of postoperative analgesia [19]. On the contrary, Bryskin et al. found that TAPB resulted in superior analgesia compared with the caudal epidural block at 6 to 24 hours after block placement, demonstrated by a statistically significant decrease in the cumulative opioid requirement [2]. Similarly, Sahin et al. showed that TAPB with a high-volume local anesthetic solution is superior to wound infiltration [8]. In the literature, there is another interesting trial comparing TAPB with quadratus lumborum block (QLB) in children undergoing unilateral inguinal hernia repair or orchidopexy, which resulted in QLB being superior to TAPB [20].

Given the fact that a low clearance because of liver function immaturity during the first year(s) of life for local anesthetics and the currently used local anesthetic is metabolized by the liver and is highly protein-bound, complications could be seen with this therapeutic regime. A recent multicenter study found the incidence of overall complications associated with TAPB in children was 0.3% [21]. More important, complications were very minor and did not require any additional interventions [21]. The current study revealed no complications during real-time US-guided TAPB in children. Besides, no block failures were diagnosed at the postoperative care unit based on inadequate analgesia.

Our results should only be interpreted within the context of their limitations. First, the analgesic effect of variable amounts of general anesthetics that presumably extend into the early postoperative period may leaded low pain scores. Second, in accordance with our daily practice, all children who received dexamethasone and paracetamol could have a positive influence on postoperative pain assessment and PONV occurrence. Finally, we could have added a control group, but it seems like we don’t expect a significant difference as good quality analgesia is achieved with this methodology.

In conclusion, this prospective, randomized controlled trial demonstrated that US-guided TAPB using 1 mg.kg^− 1^ bupivacaine administered in either high or low volume provides adequate pain control. Clinical outcomes reported here provide consistent data to support the efficacy and safety of TAPB for pain management of pediatric inguinal hernia repair.

## Supplementary Information

**Additional file 1.**

**Additional file 2.**

## Data Availability

All data generated or analyzed during this study are included in this published article as supplementary information files.
