# Supplementary material for: Transversus abdominis plane block with different bupivacaine concentrations in children undergoing unilateral inguinal hernia repair: a single-blind randomized clinical trial
Source: BMC Anesthesiol. 2022 Nov 21;22:355. doi: 10.1186/s12871-022-01907-y (PMC9677701; doi:10.1186/s12871-022-01907-y)
Supplement: Supplementary file 2 — Additional file 2. [file 12871_2022_1907_MOESM2_ESM.pdf]

The comparison of FLACC behavioral pain scores in two groups up to the first 24 hours.

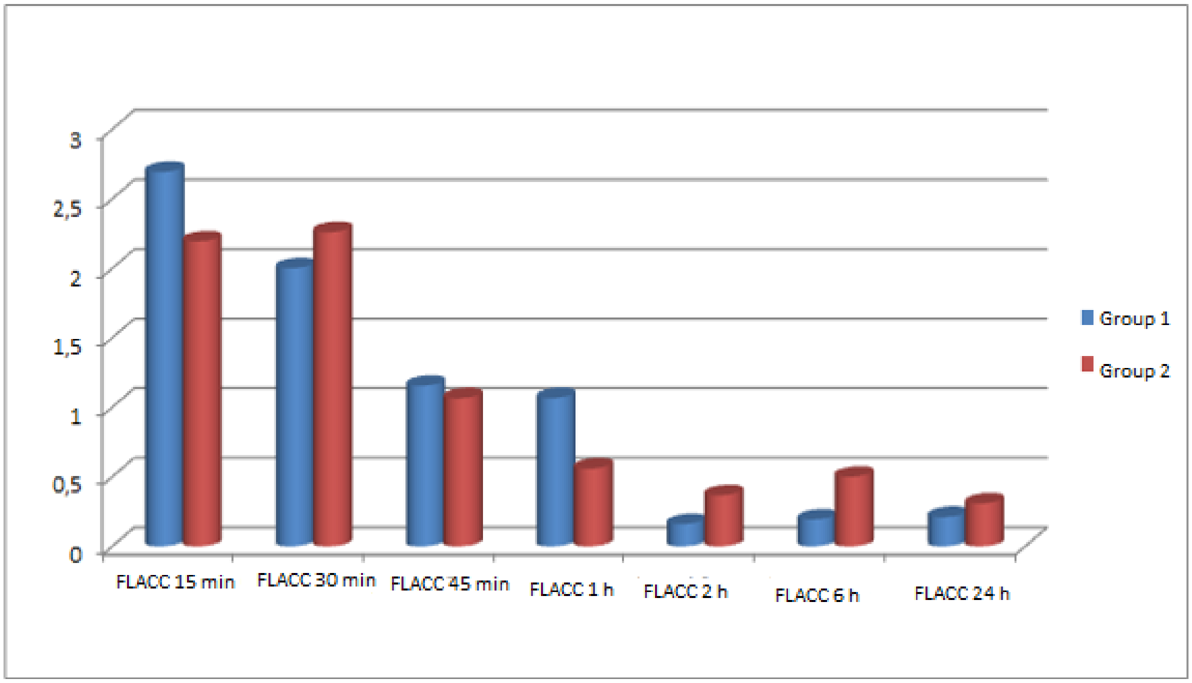

Supplementary Figure 1. FLACC behavioral pain scores are shown at 15-, 30-, 45- minutes, and 1-, 2-, 6-, 24-hour.
